# Psychosocial Factors in the Experience of Epilepsy: A Qualitative Analysis of Narratives

**DOI:** 10.1155/2021/9976110

**Published:** 2021-07-26

**Authors:** Simona Mlinar, Zvonka Rener Primec, Davorina Petek

**Affiliations:** ^1^University of Ljubljana, Faculty of Medicine, Vrazov trg 2, 1000 Ljubljana, Slovenia; ^2^University of Ljubljana, Faculty of Medicine, Department of Child, Adolescent and Developmental Neurology, Children's Hospital, University Medical Centre, Bohoričeva 20, 1000 Ljubljana, Slovenia; ^3^University of Ljubljana, Faculty of Medicine, Department of Family Medicine, Poljanski nasip 58, 1000 Ljubljana, Slovenia

## Abstract

**Introduction:**

Epilepsy is a complex disease. The consequences of epilepsy are varied and manifested in all aspects of people with epilepsy's (PWE) lives. The purpose of this study was to define individual experiences of epilepsy, expressed in narratives, and to find the stem of each narrative—a core event in the PWE's experience of the disease around which they structure their overall narrative.

**Method:**

A qualitative, phenomenological research method was used. We conducted semistructured interviews with 22 PWE and analysed the content using a combination of inductive and deductive methods, based on which we determined the stem narratives.

**Results:**

The stem narrative of the epilepsy narrative is an important life experience of PWE. We divided the stem narratives into four groups: lifestyle changes, relationship changes, the consequences of the inciting incident, and the limitations of the disease. In our study, we found that the stem narrative was, in all but one case, a secondary (psychosocial) factor resulting from epilepsy, but not its symptom (epileptic seizure). The stem narrative, where aspects of life with epilepsy are exposed, points to a fundamental loss felt by PWE.

**Conclusion:**

The narrative of the experience of epilepsy has proven to be an important source of information about the disease and life of PWE and also about the aspects at the forefront of life with epilepsy. The secondary epilepsy factors that we identified in the stem narratives were the greatest burden for PWE in all cases but one.

## 1. Introduction

Epilepsy is a complex disease. The consequences of epilepsy are varied and manifested in all areas of people with epilepsy's (PWE) life. The experience of this disease increases the intensity of living with epilepsy, which may be the basis for explaining the relationship between the internal mechanism of adaptation to the disease and a positive interpretation of the disease [1]. Epileptic seizures are unpredictable; they increase the risk of injury, hospitalisation, and even mortality. Coping with the consequences of epilepsy, especially its typical burden of the accompanying social stigma, depends on the individual's personality traits. Different episodes of discrimination, coping strategies, and personality may be important in feeling the stigma of epilepsy [2]. Stigmatisation leads to discrimination against PWE in multiple aspects of life [3].

The presence of a narrative/narration of living with epilepsy in everyday life is almost self-evident. Language is an integral part of social life [4]. The way one narrates reflects one's fundamental perception of oneself and one's relationship to others and to the world [5]. Kerby defines narrative as the design of chronological events to show their broader meaning [6]. Narrative analysis allows us to explore specific social phenomena and human fundamental sociability through specific life stories [7]. Kleinman notes that for a holistic understanding of the patient, the physician must first compile a narrative of the disease as presented to him by the patient and only then interpret it in terms of the disease's symptoms [8]. The stem narrative of the epilepsy narrative, which was the objective of our study, is an important life experience of PWE. Generally, “traditional” quantitative research does not describe the impact of epilepsy on an individual's life—within the family and in society [9]. To date, qualitative research on epilepsy has predominantly addressed individual aspects, such as the impact of epilepsy on the quality of life of PWE associated with their stigmatisation, discrimination and employment opportunities, mental disorders, lifestyle, the burden of disease, and pregnancy [10–12].

Compared to the majority population, PWE have a lower quality of life [13–15]. The impact of epilepsy on quality of life has been recognised in the literature as an important factor in the care of PWE.

Narrative studies of the experience of epilepsy are rare. In reviewing the literature, we found a single study on the narrative of PWE, which is aimed at understanding life, improving clinical practice, and providing support to PWE. Brosh studied the life of PWE in terms of the occurrence, changes, and challenges of the disease. In her narrative analysis of written reports, she highlighted five different narrative typologies. She found that the narratives reflect the way PWE make sense of their disease, how they deal with adversity, and so on [16]. Another study found that stories about the disease have a complete narrative structure: they are composed of shorter stories, which are headed by the life story of the disease [17]. Shimazono explored how storytelling enriches knowledge and understanding of disease-related experiences. According to him, narration is a special means of communication that gives meaning to a time-defined human experience [18]. Our study is based on this assumption.

The purpose of our study was to define an individual's experience of epilepsy expressed in a narrative and to search for its stem narrative—the fundamental event of the experience of the disease around which PWE design their narrative.

The protocol for conducting the research was approved in February 2012 by the Republic of Slovenia National Medical Ethics Committee (document ID number 109/01/12).

## 2. Method

### 2.1. Theoretical Framework and Research Approach

This research paper presents a narrative of PWE's experience of the disease. We labelled the core part of the narrative the *stem narrative*. We also present our technique for identifying the stem narrative.

The research method we applied is qualitative and phenomenological-based. This method allowed us to access the experiences of epilepsy through PWE and to deepen the understanding of an individual's experience through the awareness of the disease [19]. In the process of developing the research protocol, we contacted the League Against Epilepsy of Slovenia and obtained information on whether such research would be interesting for PWE. We were also interested in their opinion on which aspects of the research would be important for them and what is the biggest challenges of life with epilepsy. PWE were invited to describe an actual experience with detailed descriptions and concepts as they experience it [20]. This approach is suitable for studying lesser-known areas and exploring the mental and social dimensions of health. It is intended to accurately describe, analyse, and interpret experience from an individual's perspective conveyed in a personal story [21, 22].

### 2.2. Participants

The inclusion criteria were

- persons diagnosed with epilepsy (all forms)
- legal age of majority (in Slovenia with 18 years)
- the ability to understand and sign the consent form for participation in the study

Participants were invited by way of the “snowball sampling” method. This sampling method is suitable for studying hard-to-reach and marginalised populations, which also include PWE, due to the stigma of their disease [23]. The person who conducted the interviews (SM) sent an email invitation to PWE's relatives, colleagues, friends, and the Slovenian League Against Epilepsy, containing the basic information about the study and framework topics that covered living with epilepsy. We also asked them to send the invitation to other PWE they know or to those they think might know other PWE. We wanted the candidates themselves to express their wish to be included in the research study, to “willingly” tell their narratives. Therefore, only the persons who contacted us were included in the study. An agreement to conduct an interview followed.

#### 2.2.1. Conducting the Study

We conducted semistructured interviews with 22 PWE. All of them signed a consent form on voluntary participation in the study. We also explained to them that they could revoke their consent at any time. One of the participants decided to leave the study after the interview. She stated that the reason was her parents' fear and concern that she would be recognised as a PWE. The conversation with her was not included in the analysis moving forward. We ensured the anonymity of the participants by encrypting their personal data.

### 2.3. Interviews

The interviews with the participants were conducted between November 2011 and March 2012 at various locations across Slovenia. Participants chose the place and time of the interview themselves. Fourteen interviews were conducted at the participants' homes, five in a public place, and three on the premises of the Slovenian League Against Epilepsy.

All interviews were conducted by one person (SM). They lasted from 35 to 130 minutes. During the interviews, participants were encouraged to talk about epilepsy as spontaneously as possible and to highlight important events for them. We did not limit the length of the interview, because we did not want to limit the participants or make them feel uncomfortable in case of “longer” narrations, since imposed time restrictions could affect the data completeness [24]. We recorded the interviews, transcribed the recordings, and compared the accuracy of the transcripts with the original audio recordings. We also collected the participants' basic sociodemographic data (Appendix 1).

### 2.4. Analysis

The purpose of the analysis was to find the event in the experience that had (or has) an essential impact on PWE. We labelled it the *stem narrative*.

We performed a content analysis of the texts. The analysis unit was the narrative of the disease (the entire interview), as it was only possible to decipher the stem narrative through the entire narrative. This was followed by defining the stem narrative, encoding the text, and setting the criteria that represent the common characteristics of stem narratives. The analysis was carried out using a combination of inductive and deductive methods. We used the inductive method to set the criteria within the stem narrative and the deductive method for encoding further interviews according to the previously set criteria.

The interviews were transcribed in Microsoft Word. Two persons (SM and DP), independently of each other, first conducted a pilot analysis of three interviews in order to find their stem narratives. Afterwards, they unified their findings. Determining the stem narrative was the first act of analysis and the starting point for the interpretation of the narrative, i.e., the event that shaped the life of PWE. They then extracted the stem narrative from the rest of the interviews in the same way as in the pilot analysis. According to the inductive method and the set stem narrative, the text was encoded to set the criteria for the first three interviews. The text was encoded by two people, independently of each other, according to the previously set stem narrative, and unified at the end of the encoding process. We looked for parts of the narrative in the text that are related to the experience recognised as the stem narrative. The encoding unit was represented by a smaller content unit, e.g., paragraph/short text. It described an individual aspect of the experience and alluded to the stem narrative.

**Figure 1:**
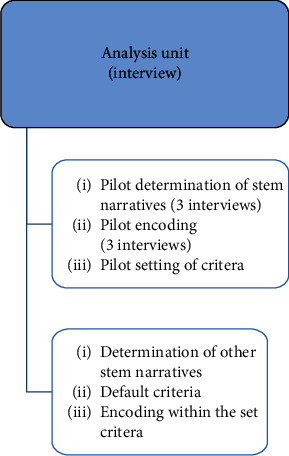
Analysis overview.

The encoding process was open: the codes came from the text and were written into the text during reading, axially (some codes belonged to different higher categories) and selectively (according to the analysis matrix). Similar codes were combined in a meaningful way and, according to their connection with the stem narrative, classified into categories that represent their common qualities. We named the categories “criteria.” The number of codes by stem narrative for each criterion is presented in Appendix 3. Each criterion represented a particular aspect of the stem narrative; they represented the analysis matrix. The remaining interviews were analysed using the deductive method according to the previously set criteria. In the interview analyses, we allowed for the possibility of setting additional criteria. The additional criterion was the cause of stem narrative, which appeared in the fewest stem narratives. Everything was recorded and transcribed in Microsoft Word.  Figure 1 (“analysis overview”) represents the content analysis of the text.

## 3. Results

### 3.1. Sociodemographic Data

Twenty-one PWE were included in the analysis and the study, out of which 14 were women and 7 were men. Their age ranged from 27 to 66 years (mean age is 46.9 years). The mean age of PWE at the time of the first seizure was 17.0 years. The earliest first seizure of any of the participants was at the age of five years, and the latest was at the age of 43 years. Ten PWE were married, two had partners, and nine were single. Most PWE had completed secondary school (twelve of them), six of them had completed primary school, and three had completed college. Eight PWE were employed, five were unemployed, six were receiving a pension, one person lived on an annuity, and one participant was a monk and lived in a monastery. The shortest time since the last seizure was two days before the interview, and the longest was 20 years without a seizure. One of the participants did not remember when his last seizure occurred. All PWE were receiving therapy at the time of the interview. Eight of the 21 participants were members of the Slovenian League Against Epilepsy.

### 3.2. The Stem Narrative

The following stem narratives^∗^ were identified through analysis.

Based on selective encoding according to the stem narratives of the three interviews, we set the following criteria to describe the stem narrative:

- The emotional aspect
- Personal engagement
- The consequences
- Its connection to relationships
- The cause
- Referencing (returning back to) frequency of the event we identified as the stem narrative

These criteria defined the content of each stem narrative. They provide a basis for assessing why a particular experience is recognised as a stem narrative. Not every one of them contains all of the criteria. The content of individual criteria sometimes overlaps. The content of each criterion is summarised in the table of subcategories and their associated codes by criteria (Appendix 2). The subcategories and codes in the criteria may be the same in name but substantively different depending on the uniqueness of the stem narrative and the circumstances of the event that represents it. The stem narratives were divided into four areas based on their content, as shown in Table 1.

#### 3.2.1. Lifestyle Change

PWE have often pointed out that self-observation is an important cognition. They described this in words: *to know each other*, *to get to know each other*, *to know oneself*, *to say things about oneself*, *etc*. In addition to the changes brought about by epilepsy, we included PWE's own efforts to improve, change, or influence the consequences of an event (experience) that was defined as the stem narrative. All PWE have in common trying to make sense of their “new” condition after the confirmed diagnosis.

PWE recognised knowing their own body's responses as important. To improve life with epilepsy and control the disease, its management in everyday life must be based on good knowledge of the body's responses and the impact of various mental states on the body. The change manifests itself in adapting life to the disease.

*If I don't listen to myself when I'm not feeling well, I'm going to have a seizure. I have to pay attention to when I feel fine and when I have to stop. I know exactly what it is that can cause me to have a seizure. An epileptic knows what threatens him. What can provoke a seizure and what to avoid. But he also knows how to protect himself from this happening, because he knows himself so well.* (M.1)

The disease brought discipline and order to everyday life. PWE adjusted their daily routine, taking therapy, leisure activities, spending time with their family, etc. PWE pay considerable attention to everyday tasks. Daily burdens are associated with a certain amount of stress. The interviewees believed that stress was the most common trigger of an epileptic seizure.

*Nerves, stress is the most common. I attribute three-quarters of the cause* (for the seizure, A/N) *to nerves. We have a business and it is very difficult sometimes. Problems need to be put aside and I need to live as calmly as possible. But this is not feasible. I have let go of many things, but to leave everything and to live anew is impossible.* (W.12)

PWE intentionally reduce stress and nervous tension with indifference, detachment, and “intentional unencumberedness.”

*Like I said, if you have this disease, you shouldn't stress yourself. If we cleaned, great, but if we didn't, it was as it was. If I have a seizure, I can't clean the house, so it stays the way it is. Maybe someone will tell me that I'm not normal, but because of my health, I have to leave it.* (W.13)

PWE strive for autonomy and independence. The event, identified as the stem narrative (in Table 2), brought change to the lives of PWE. This change is not always negative, but PWE experienced it more intensely.

#### 3.2.2. Relationship Changes

All of the stem narratives have a relational component. PWE are most emotionally affected when it comes to accepting or not accepting themselves. They describe with strong emotion the ignorance regarding epilepsy shown by public institution employers, coworkers, and employees. An important aspect of the emotional and relational dimension of the stem narrative is the time after diagnosis. In PWE, strong feelings of anger, despair, and insecurity are intermixed during this period. More frequent epileptic seizures further intensify these emotions.

Stem narratives, positive or negative, markedly encroach on relationships. PWE find that epilepsy makes it easier to accept other people and better understand other patients. One of the interviewees changed her view of others after her own experience with limitations due to epilepsy.

*There are absolutely some advantages* (disease, A/N). *You yourself have a certain limitation, a certain problem and you know how to accept other people through it. You look at people with difficulties, problems and needs differently, because you also have certain problems. You identify with them because you have a problem yourself and you try to help them because you care.* (M.1)

All stem narratives involve relationships with parents, siblings, partners, relatives, children, coworkers, and employers. Relationships reflect the burden of epilepsy: nonunderstanding, limitations at work, lifestyle changes, physical helplessness after a seizure, various forms of fear, the impact on social security, hiding the disease, and so on. The environment in which this PWE lives and the relationships she has established have both shaped her (or their) narrative of the experience of epilepsy.

*It was taboo for my parents. No one knew I had epilepsy, they didn't even know it in school. My parents told the school I had heart problems. In case I accidentally fell unconscious. For my mother, this is still taboo today.* (W.18)

*My mother is a fighter and she never pitied me or wrapped me up in cotton wool because of the disease. She always instilled in me that I have a such and such disease, which needs to be accepted and lived with. As far as that goes, she equipped me very well. I accepted that.* (M.1)

PWE have varied relationship experiences with relatives. For some, caring for relatives is burdensome. Unemployed PWE are financially dependent on their relatives. They have a sense of guilt. PWE often do not want the help of relatives and want to remain independent. Family members help them integrate into society, while they are confronted with the unpredictability of their loved one's epilepsy and its consequences. After the diagnosis is made, family dynamics, the roles of other family members, and the distribution of responsibilities among family members change.

*They left me alone for two, three months, so I had no problems. I really had too much of everything before ... After my husband and sons also saw what it was all about, it was much easier. I kind of forgot I had it* (epilepsy, A/N). *I have come to terms with the fact that it is as it is. We need to move forward.* (W.12)

Relationships are particularly affected by the disclosure of the disease. There is a certain amount of discomfort, fear, and doubt about the response of the person to whom PWE reveal their disease. The identification of the stem narrative in our study proves that a key change in the experience of the disease, as well as in the closer (partner, family) and more distant social relationships, occurred at the time of disclosure/personal exposure.

*Hurt. Lonely. You feel very alone in this world. People are unsympathetic, you can't be accessible to them because if you are accessible to them and you tell them things like that, they dismiss you. It's a very terrible thing ... It's not an easy thing.* (W.8)

#### 3.2.3. Consequences of the Stem Narrative

The event that we identified as the stem narrative is not always equal to the burden of epilepsy. The stem narrative can be closer to the consequences of epilepsy and can completely overlap it. This experience, recognised as the stem narrative, shapes the life of PWE at that moment and influences their future. The inciting incident does not only have negative repercussions; however, they prevail.

*I can't say that I was really scared, but epilepsy changed my lifestyle. Humans contemplate a lot. You don't work anymore, I was a communicative person, in society and after this thing* (epileptic seizure, A/N) *happens, the way of life or the way of thinking about life actually changes.* (M.14)

According to some PWE, their lives would not have developed as they did without the emotions, physical awareness, etc., represented by the inciting incident. One of the interviewed PWE placed an event at the centre of the narrative; he first assessed it as negative and later recognised it as significant.

*That's why I thought about this thing* (disease, A/N) *mostly in a spiritual sense. I somehow began to understand the story of my illness as a wonderful gift from god. With this illness, god pressed me so hard against the ground that I had to forget all of my plans. I then understood that this illness is a wonderful signpost from god, so I was never sad because I was ill, but I was glad to have the gift of this disease ... When you look back, it becomes clear to you.* (M.15)

The inciting incident is largely an emotional burden. PWE most often expose stressful experiences in the event of the death of a loved one, divorce or departure of a partner, loss of employment, unemployment, etc. PWE who accepted their disease no longer cultivated negative feelings towards themselves or others.

#### 3.2.4. Limitations of the Disease

PWE most often point out difficulties in finding a job as a limitation of the disease: the employer does not have a suitable job for them, withholding their diagnosis due to fear of unemployment and ignorance about epilepsy, their relationship with the employer and coworkers. One of the PWE (stem narrative 18) did not find sympathy with any of the institutions she approached to regulate her status. She perceived the core of her problems in the set limitations of epilepsy. Because she felt fine, she was convinced that the set limitations were too general and did not correspond to her health status.

*I always told* (that I have epilepsy, A/N). *The responses, however, were that they don't need you. I even had an HR employee tell me how dare I look for a job, given that there are so many healthy people looking for a job. I said I need a job too, because I have a family too ... I would love to do anything. At a medical examination, they gave me a list of everything I shouldn't do. There is almost no work left for me to do.* (W.18)

One of the biggest challenges and ambitions of PWE is to have suitable employment. PWE find it difficult to find a suitable job. In their narratives, they emphasise that once they were employed, they performed the work with diligence and enthusiasm.

## 4. Discussion

Our study found that an individual's experience with epilepsy affects his or her life in four important areas: lifestyle changes, relationship changes, the consequences of the inciting incident, and disease limitations. In the study, we found that the stem narrative was, in all but one case, a secondary (psychosocial) factor that resulted from epilepsy but not its symptom (epileptic seizure). Similarly, when Batchelor and Taylor examined relationships between psychosocial variables (coping strategies and sources of social support) and mental health outcomes, they noted that psychosocial factors impact mental health in PWE whereas seizure-related factors do not [25]. The stem narrative, in which the negative aspects of life are exposed, points to a fundamental loss felt by PWE due to epilepsy. Regardless of the success of epilepsy treatment, the social and personal adaptation of PWE to the disease is a key challenge in many, if not all, areas of life [26]. Positive coping strategies (such as problem-focused coping, faith in God, social support, and lifestyle changes) lead to better health outcomes [27]. The better PWE know themselves with the disease, the easier it is for them to live with it.

PWE shaped their overall narrative around the stem narrative [28]. They interpreted their life with epilepsy through the stem narrative. They spontaneously returned to it several times during their narration. This finding is confirmed by Mishler, who finds that an individual's impulse to tell is such a fundamental part of the human experience that interviewees will tell a story/events about their lives, even if they are not encouraged to do so [29].

The stem narrative that we analysed in the conducted research and the criteria that we developed do not have a parallel in the reviewed literature. A study of PWE narration by Brosh sought to develop understanding, improve clinical practice, and strengthen PWE support through diagnosis in adulthood. Her narrative analysis showed that epilepsy affects individuals differently, at different times, and in different contexts [16]. Her findings can be extended by the fact that epilepsy in different individuals defines their lives and the lives of their families differently. We found that the lives of various individuals and their families are not only marked by epilepsy but that this disease has a profound effect on family relationships or social components of life. The stem narrative speaks to the different dynamics of relationships at all levels.

A person diagnosed with epilepsy first devotes most of their time to dealing with the disease. They often lack information on assistance services [12]. They change their habits. They no longer perform certain tasks. They cease certain activities and duties. Their loved ones sometimes cannot accept this or often do not understand it. The situation is further complicated by the long-term or permanent condition of the situation. Both medical and psychosocial factors have an important impact on the psychosocial functioning of PWE. The findings of Clarke and Critchley suggest that clinicians may promote better outcomes by strengthening family functioning and encouraging the use of productive (emotion-focused) coping strategies [30]. The efforts of PWE and the encouragement of their families are not negligible. This is confirmed by Vaccarella in *The Lancet*. According to her, neurologists and family physicians are aware that successful treatment of epilepsy goes far beyond addressing epileptic seizures and comprehends inclusion and acceptance in one's living environment [26].

The stem narratives showed that epilepsy brings new dynamics to partner relationships, relatives and family, and the living environment (separation, loneliness, loss of autonomy, hiding the disease, etc.). Developing epilepsy represents a significant event in individuals' lives [31]. The stem narratives demonstrated how the environment perceives PWE (burden of the disease, deprivation of autonomy, care from relatives, etc.). This relational level was strongly influenced by the disclosure of the disease. Espinola-Nadurille and others noted that PWE have experienced the departure of a partner or loved one due to their disease or after they witnessed an epileptic seizure [32]. PWE's expectation in the study, that disease disclosure will be more effective in relationships, has not always been met. Even those who revealed the disease to their loved ones voluntarily faced rejection, and this often led to their isolation and withdrawal. Neighbours expressed their rejection indirectly, i.e., as a doubt that they will not be able to respond appropriately in the event of a seizure. Our finding is consistent with the results of Baker's research, stating that isolation and withdrawal of PWE are common consequences of anxiety due to hostile responses to epileptic seizures in public [33].

The relational level of stem narratives also involves the need for PWE to assist others. Our research shows that PWE are aware of the help of others in their daily lives, including financial and social support. In addition to providing assistance to relatives, we also detected that PWE feel a burden. It is linked to the autonomy of PWE if their relatives take full care of them, make decisions for them, and set boundaries for them. Similarly, Švab notes for people with mental health problems that feelings of fear, compassion, sadness, and insecurity often encourage discriminatory behaviour by family members and professional staff. The most common defensive response to these emotions is the patronising relationship of family members and staff [34].

The inciting incident of the stem narrative was one of the key reasons why PWE decided to present/tell their story of the disease. An inciting incident does not always express the disease directly. In the stem narrative, we have often recognised the need for PWE to express disagreement, injustice, or hurt about an individual event related to the circumstances and consequences of epilepsy. We saw this mostly in the negative consequences of the stem narrative; however, this did not apply to all interviewees. All interviewed PWE have attributed stem narratives with negative consequences to epilepsy. They thought that a disease-free situation (social, financial, relational, etc.) would be different or better. Based on these findings, we agree with Admi and Shaham that epilepsy is not at the heart of PWE's daily life and that methods of coping with the disease are adapted to the situation in terms of social stigma and practical possibilities [10]. Finding meaning in the disease, dealing with it, and accepting forthcoming changes in the life of PWE are all influenced especially by one's own efforts. Michaelis and others came to similar conclusions by suggesting that the patients' development of self-efficacy was motivated by their personal initial goals and facilitated by the encouragement to find individual solutions [35].

In the stem narratives, we detected various limitations that PWE experience in everyday life. Most often, they had difficulty finding employment and maintaining a romantic relationship. They experienced unemployment or loneliness as an injustice that has happened to them. Employment is an area where PWE's personal efforts stand out. Unemployed PWE made great efforts to obtain a job, while employed PWE emphasised the diligence and independence in performing their work. The correctness of this finding is supported by the research conducted by Jacoby and others, stating that the employer's fear is the most common obstacle that prevents them from employing PWE. PWE thus express the angst of being discriminated against and stigmatised at their work [36]. Work gives people a sense of identity, who they are, what they can do, and what their role in society is. It is a source of relationships outside the family. As a rule, unemployment not only reduces social contacts but also cripples them in the family [37]. Our research shows that PWE are the least successful in coping with job search difficulties. The importance of working is not only about financial independence and autonomy but also about understanding the position of PWE within the family.

Stem narratives recount how PWE feel in a certain environment, how they are integrated into it, what they miss, or what will improve their status and inclusion in a wider environment. This is also confirmed by Dibell with the fact that narration (designing the narrative) is a way of looking at things. By connecting the events in the narrative, the individual shows how important things are to him in the immediate environment [38].

## 5. Limitations of the Study

Our study had limitations that we considered in the interpretation of our findings.

1. The type of epilepsy and its course did not affect the criteria for inclusion in the study. Some forms of epilepsy have more severe and long-lasting consequences, and the burden of the disease in these individuals is greater [39]. Although the characteristics of the disease were not evaluated in the PWE included in the study, it is evident from the conversations that PWE with various forms of epilepsy were included. Sampling in the qualitative research was intentional and occasional, but not random, so that the sample was not representative of the population, and therefore, the findings of the study cannot be generalised to the entire population of PWE. However, our sample was very diverse according to many criteria, with which we wanted to gain a wide range of experience with the disease. Open coding revealed that no new codes emerged from the text and the codes started to repeat themselves. Therefore, we can conclude that the number and extent of interviews and the richness of the data allow us to claim data saturation
2. The next limitation concerns determining the stem narrative. Although we encouraged PWE to narrate their illness as independently as possible, this was not always possible due to various problems (e.g., memory) and aspects of the disease. Therefore, it is possible that we directed the narrative by encouraging them and thus inadvertently influenced the content of the narrative
3. We also state a limitation regarding the phenomenological design of the study. Although one of the strengths of phenomenology is the focus on exploring a specific experience, it does not allow for a generalisation of event identification for an individual or group of individuals [40], in our case for PWE. With the help of phenomenological design, we can (only) describe what it is like to have a certain experience

## 6. Conclusion

The uniqueness of human life with a disease is incomparable. The importance of context in the narrative stands out and emphasises the uniqueness of the individual. Each narrative of PWE is unique because of the uniqueness of the narrator. No two narratives of the epilepsy experience were the same, even though the stem narratives may have been designated under the same title. The stem narrative represents a kind of deeper existential core that demonstrates, directs, connects, influences, and illuminates how PWE experience social inclusion. The narrative of the experience of epilepsy has proven to be an important source of information about the disease and life of PWE, as well as aspects that are at the forefront of life with epilepsy. Our research showed that secondary factors of epilepsy are the greatest burden for PWE in all but one case.

The patient's life and experience of the disease take place on several levels. In addition, chronic diseases, where epilepsy is no exception, are associated with a permanently changed lifestyle; therefore, it is not always clear what the cause is and what the consequence is. Changes occur in the physical, mental, interpersonal, and social spheres. Patients learn to live with the disease. They incorporate the changes brought about by the disease into their lives. Knowledge of the experience of the disease is important, whereby patients can develop appropriate behavioural approaches to manage their disease and prevent more severe consequences. Defining the stem narrative of the experience of the disease therefore constitutes a key contribution to understanding the needs required for more complex, comprehensive, and structured treatment of PWE.

## Figures and Tables

**Table 1:** Stem narratives according to area^∗^.

	Area	Stem narrative
(1)	Lifestyle change	6, 9, 12, 13, 14
(2)	Relationship changes	1, 3, 5, 11, 17, 19, 21
(3)	Consequences of the inciting incident	4, 7, 10, 16, 20
(4)	Disease limitations	2, 8, 15, 18

^∗^Stem narrative numbers are divided into areas according to their content.

**Table 2:** Determined stem narratives.

(1) A changed view of yourself and others (M)^∗∗^
(2) Unemployment due to disease limitations (W)
(3) Loss of independence and burden of relatives' care (W)
(4) Nonunderstanding of the burden of disease at school and work (W)
(5) The relationship and role of the husband (W)
(6) Giving up sports activities and separation from the partner (M)
(7) Alcohol addiction (M)
(8) Limited work ability and effort in performing work (W)
(9) Giving up further education (W)
(10) Loneliness (W)
(11) Concealment of the disease (W)
(12) Lifestyle adjustment (W)
(13) Accepting the disease and lifestyle change (W)
(14) Lifestyle change (M)
(15) Career change (M)
(16) Physical hazard during an epileptic seizure (W)
(17) Difficult family situation (W)
(18) Disability and difficulty retiring (W)
(19) The burden of the disease in a romantic relationship (W)
(20) Efforts to keep a driving license (M)
(21) Initial concealment and subsequent disclosure of the disease (M)

^∗^The reference for each number represents the stem narrative of one interview. ^∗∗^Letter W/M represents whether PWE (interviewee) is male (M) or female (F).

## Data Availability

Access to data is restricted. Access to the data is not possible due to the protection of data on research participants. As part of the research, we assured the participants that data on their health status and experience of the disease will be published in an anonymized form.

## Supplementary Materials

Supplementary Materials Appendix 1: sociodemographic data of PWE included in the study. The table shows the PWE data included in the survey. We collected data on the age of PWE, marital status, environment in which PWE lives, education, employment, age at the first epileptic seizure, time elapsed since the last epileptic seizure, and whether PWE is included in the League Against Epilepsy of Slovenia. Appendix 2: subcategories and their associated codes by criteria. The table shows the individual criteria and their subcategories. We included in the subcategories the codes that we defined during coding. Subcategories with codes show the content of each criterion. Appendix 3: number of codes by stem narrative for each criterion. The table shows the number of codes in each criterion according to each stem narrative. The number of codes indicates the frequency of their occurrence in a particular criterion.

## References

[1] Büssing A., Ostermann T., Neugebauer E. A. M., Heusser P. (2010). Adaptive coping strategies in patients with chronic pain conditions and their interpretation of disease. *BMC Public Health*.

[2] Sang-Ahm L., Hee-Jung Y., Byung-In L. (2005). Factors contributing to the stigma of epilepsy. *Seizure*.

[3] Pahl K., de Boer H. M. (2005). *Epilepsy and Rights. V: Atlas: Epilepsy Care in the World*.

[4] Fairclough N. (2003). *Analysing Discourse. Textual Analysis for Social Research*.

[5] Elwyn G., Gwyn R., Greenhalg T., Hurwitz B. (2004). Stories we hear and stories we tell: analysing talk in clinical practice. V. *Narrative Based Medicine*.

[6] Kerby A. (1991). *Narrative and the Self*.

[7] Chase S. E., Denzin N. K., Giardina M. D. (2008). Taking Narrative Seriously: Consequences for Method and Theory in Interview Studies. V. *The Elephant in the Living Room, OR Advancing the Conversation about the Politics of Evidence. Qualitative Inquiry and the Politics of Evidence*.

[8] Kleinman A. (1989). *The Illness Narratives. Suffering, Healing and the Human Condition*.

[9] Bishop M., Allen C. A. (2003). The impact of epilepsy on quality of life: a qualitative analysis. *Epilepsy & Behavior*.

[10] Admi H., Shaham B. (2007). Living with epilepsy: ordinary people coping with extraordinary situations. *Qualitative Health Research*.

[11] Jacoby A., Ring A., Whitehead M., Marson A., Baker G. A. (2014). Exploring loss and replacement of loss for understanding the impacts of epilepsy onset: a qualitative investigation. *Epilepsy & Behavior*.

[12] Chunk K., Liu Y., Ivey S. L. (2012). Quality of life in epilepsy (QOLIE): insights about epilepsy and support groups from people with epilepsy (San Francisco Bay Area, USA). *Epilepsy & Behavior*.

[13] Norsa’adah B., Zainab J., Knight A. (2013). The quality of life of people with epilepsy at a tertiary referral centre in Malaysia. *Health and Quality of Life Outcomes*.

[14] Kotsopoulos I. A., Evers S. M., Ament A. J. (2003). The costs of epilepsy in three different populations of patients with epilepsy. *Epilepsy Research*.

[15] Tellez-Zenteno J. F., Pondal-Sordo M., Matijevic S., Wiebe S. (2004). National and regional prevalence of self-reported epilepsy in Canada. *Epilepsia*.

[16] Brosh L. (2011). *Narratives of living with epilepsy diagnosed in adulthood*.

[17] Good B. J., del Vecchio Good M. J., Togan I., Ilbars Z., Güvener A., Gelişen I. (1994). In the subjunctive mode: epilepsy narratives in Turkey. *Social Science & Medicine*.

[18] Shimazono Y. (2003). *Narrative Analysis in Medical Anthropology*.

[19] Giorgi A. (2009). *The Descriptive Phenomenological Method in Psychology*.

[20] Adams C., van Manen M., Given L. M. (2008). Phenomenology. V. *The Encyclopaedia of Qualitative Research Methods*.

[21] Thompson E. (2007). *Mind in Life. Biology, Phenomenology and the Sciences of Mind*.

[22] Škodlar B. (2008). *Phenomenological Analysis of Reasons for Suicide in Schizophrenia Patients: Doktorska Disertacija*.

[23] Scambler G., Hopkins A. (1990). Generating a model of epileptic stigma: the role of qualitative analysis. *Social Science & Medicine*.

[24] Mack N., Woodsong C., MacQueen K. M., Guest G., Namey E. (2005). *Qualitative Research Methods: A Data Collector’s Field Guide*.

[25] Batchelor R., Taylor M. D. (2021). Young adults with epilepsy: relationships between psychosocial variables and anxiety, depression, and suicidality. *Epilepsy & Behavior*.

[26] Vaccarella M. (2020). *The Art of Medicine. Narrative Epileptology*.

[27] Ochs E., Duranti A. (2006). Narrative Lessons. V. *A Companion to Linguistic Anthropology*.

[28] Deegbe D. A., Aziato L., Attiogbe A. (2020). Experience of epilepsy: coping strategies and health outcomes among Ghanaians living with epilepsy. *Epilepsy & Behavior*.

[29] Mishler E. G. (1986). *Research Interviewing. Context and Narrative*.

[30] Clarke A. L., Critchley C. (2016). Impact of choice of coping strategies and family functioning on psychosocial function of young people with epilepsy. *Epilepsy & Behavior*.

[31] Rawlings G. H., Brown I., Stone B., Reuber M. (2017). Written accounts of living with epilepsy: a thematic analysis. *Epilepsy & Behavior*.

[32] Espinola-Nadurille M., Crail-Melendez D., Sanchez-Guzman M. A. (2014). Stigma experience of people with epilepsy in Mexico and views of health care providers. *Epilepsy & Behavior*.

[33] Baker G. A. (2002). The psychosocial burden of epilepsy. *Epilepsia*.

[34] Švab V., L’Abate L. (2012). Stigma in mental disorders. V. *Mental Illnesses - Understanding, Prediction and Control*.

[35] Michaelis R., Niedermann C., Reuber M., Kuthe M., Berger B. (2018). " Seizures have become a means of somehow learning things about myself" -- a qualitative study of the development of self-efficacy and mastery during a psychotherapeutic intervention for people with epilepsy. *Epilepsy & Behavior*.

[36] Jacoby A., Gorry J., Baker G. A. (2005). Employers' attitudes to employment of people with epilepsy: still the same old story?. *Epilepsia*.

[37] Fagin L., Little M. (1984). *The Forsaken Families*.

[38] Dibell A. (2016). *On Plot: The Elements of Fiction Writing*.

[39] Gaitatzis A., Sisodiya S. M., Sander J. W. (2012). The somatic comorbidity of epilepsy: a weighty but often unrecognized burden. *Epilepsia*.

[40] Mayoh J., Onwuegbuzie A. J. (2015). Toward a conceptualization of mixed methods phenomenological research. *Journal of Mixed Methods Research*.

