# Supplementary material for: Psychosocial Factors in the Experience of Epilepsy: A Qualitative Analysis of Narratives
Source: Behav Neurol. 2021 Jul 26;2021:9976110. doi: 10.1155/2021/9976110 (PMC8331301; doi:10.1155/2021/9976110)
Supplement: Supplementary Materials — Appendix 1: sociodemographic data of PWE included in the study. The table shows the PWE data included in the survey. We collected data on the age of PWE, marital status, environment in which PWE lives, education, employment, age at the first epileptic seizure, time elapsed since the last epileptic seizure, and whether PWE is included in the League Against Epilepsy of Slovenia. Appendix 2: subcategories and their associated codes by criteria. The table shows the individual criteria and their subcategories. We included in the subcategories the codes that we defined during coding. Subcategories with codes show the content of each criterion. Appendix 3: number of codes by stem narrative for each criterion. The table shows the number of codes in each criterion according to each stem narrative. The number of codes indicates the frequency of their occurrence in a particular criterion. [file 9976110.f1.zip › Appendix 1_sociodemographic data.docx]

Appendix 1: Sociodemographic data of PWE included in the study

| **PWE** | **Age** | **Marital status** | **Living situation** | **Education** | **Employment** | **Age at onset of epilepsy (first seizure)** | **Time since last seizure** | **Member of the Slovenian League Against Epilepsy** |
| --- | --- | --- | --- | --- | --- | --- | --- | --- |
| M.1 | 27 | single | with parents | secondary school | employed | 13 let | 2 days | No |
| W.1 | 31 | in a relationship | with partner | secondary school | employed | 16 let | 5 years | No |
| W.2 | 39 | single | alone | secondary school | employed | 19 let | 3 days | No |
| W.3 | 40 | single | alone | university | employed | 6 let | 6 years | No |
| M.2 | 59 | single | with sister’s family | primary school | early retirement due to illness | 18 let | 2 weeks | No |
| W.4 | 50 | married | with family | primary school | unemployed | 22 let | 2 days | No |
| W.5 | 40 | married | with family | secondary school | early retirement due to illness | 14 let | 5 days | No |
| W.6 | 51 | married | with family | secondary school | early retirement due to illness | 28 let | 1 month | No |
| M.3 | 50 | single | alone | secondary school | unemployed | 7 let | 12 years | Yes |
| W.7 | 64 | single | with sister | primary school | Lives off a trust fund | 13 let | Does not remember | Yes |
| W.8 | 31 | single | with father | primary school | employed | 29 let | 4 months | Yes |
| M.4 | 53 | married | with family | secondary school | unemployed | 18 let | 4 months | No |
| W.9 | 43 | married | with family | secondary school | employed | 6 let | 3 years | Yes |
| W.10 | 44 | married | with family | secondary school | employed | 42 let | 6 months | No |
| W.11 | 40 | alone | with parents | primary school | employed | 10 let | 3 months | Yes |
| M.5 | 50 | single | in a community | university | monk | 5 let | 15 years | No |
| W.12 | 42 | married | with family | secondary school | unemployed | 16 let | 2 years | Yes |
| M.6 | 66 | married | with family | university | retired | 43 let | 10 years | No |
| W.13 | 59 | married | with family | primary school | retired | 12 let | 10 years | Yes |
| M.7 | 60 | in a relationship | alone | secondary school | retired | 9 let | 20 years | Yes |
| W.14 | 46 | married | with family | secondary school | unemployed | 11 let | 1 month | No |
