# Supplementary material for: Psychosocial Factors in the Experience of Epilepsy: A Qualitative Analysis of Narratives
Source: Behav Neurol. 2021 Jul 26;2021:9976110. doi: 10.1155/2021/9976110 (PMC8331301; doi:10.1155/2021/9976110)
Supplement: Supplementary Materials — Appendix 1: sociodemographic data of PWE included in the study. The table shows the PWE data included in the survey. We collected data on the age of PWE, marital status, environment in which PWE lives, education, employment, age at the first epileptic seizure, time elapsed since the last epileptic seizure, and whether PWE is included in the League Against Epilepsy of Slovenia. Appendix 2: subcategories and their associated codes by criteria. The table shows the individual criteria and their subcategories. We included in the subcategories the codes that we defined during coding. Subcategories with codes show the content of each criterion. Appendix 3: number of codes by stem narrative for each criterion. The table shows the number of codes in each criterion according to each stem narrative. The number of codes indicates the frequency of their occurrence in a particular criterion. [file 9976110.f1.zip › Appendix 2_Subcategories and their associated codes by criteria.docx]

Appendix 2: Subcategories and their associated codes by criteria

| **Criteria** | **Subcategories** |
| --- | --- |
| **Emotional aspect of stem narrative** | - EMOTIONS (helplessness, loneliness, mistrust, desire for fatherhood, shame, feeling burdened by family, fear of seizure, insecurity, hurt, guilt, fear of the impact of illness on children, hurt and grief due to illness, coping with fear of driving, stress due to difficult working conditions, joy of work, hope for the future, handing over the future to a higher power, faith as a basis for understanding the past, feeling of non-understanding by others, affected by the opinion of the future employer, difficult living conditions, depression, difficulty of change, long-term adjustment to the disease, non-shame of disease, need for employment) - RELATIONSHIPS (separation from partner, introversion, detachment, loss of autonomy, encouragement of family, protective relationship of partner, support of partner, rejection of partner due to illness, difficult home situation, unencumbered by caring for family, fear of teachers with inappropriate attitude, awareness of non-understanding of the disease in others, awareness of poor attitude of co-workers, impairment due to lack of understanding at work, warning the employer of the poor attitude of co-workers towards PWE, unencumbered with homework, unencumbered by accepting others, downside of introversion, shock due to doctor's response) - THE BURDEN OF DISEASE (helplessness, dependence on others, burden of other people's worries, relativisation of the severity of the disease, hope of no seizure, questioning the future of the disease, early retirement, accepting memory problems, risk in life, awareness of stress, non-understanding of reasons for hiding the disease) - LIMITATIONS (acceptance of limitations, giving up leisure activities, grief due to limitations in life, non-understanding and ignorance of the disease by the employer, giving up hobbies due to financial difficulties) |
| **Personal engagement** | - DISEASE DISCLOSURE (disease concealment strategy, disease disclosure, personal explanation, disease concealment, control over disclosure, influence of circumstances on disclosure, ethical dilemma of concealing information from the employer) - INDEPENDENCE (self-confidence, belief in work ability, financial independence, belief in equality with others, calmness, order of life, desire for a certain profession, desire to help others, striving for independence, illness as a stimulus for a positive worldview, awareness of work requirements, awareness of the importance of employment, belief in the future, striving to complete education, desire to obtain a higher education, acquiring a new profession, will to change, perseverance in life, awareness of the importance of one's own will, great desire for employment, effort and persistence of PWE in work retirement) - DISEASE CONTROL (searching for information about the disease, successfully coping with the consequences of seizures, building self-esteem, being active in life, working on oneself and self-observation, observing limitations, knowing oneself, accepting the consequences of the disease, discipline in everyday life, urgency for stress-free living, adaptation to the disease, activity-schedule-day-planning, positive impact of change on life, hobbies – required to be adapted to the disease, care for medication supply, knowledge of one's own limitations, accepting the disease and not stressing about it, positive thinking, healthy well-being, the importance of outlook on life, striving to maintain balance, striving for a calm life) |
| **Consequences of stem narrative** | - BURDEN OF DISEASE (fear of inheriting the disease, strategy of hiding the disease, seizures in public, PWE loss of autonomy, self-burdening, burden of illness for relatives, weakness at the time of seizure, early retirement due to illness, introversion, inability to work, revocation of driver's license, stigma, fear of the future, hiding illness for fear of losing a job, limitations in life, awareness of the burden of disease, lying because of a favourable medical certificate) - EMPLOYMENT AND EDUCATION (difficulties in finding a job, giving up further education, inability to obtain additional education, positive aspects of employment on the well-being of PWE, decrease in workload, teacher non-understanding, employer non-understanding, unsuitable working environment, difficulty in performing work, unsympathetic attitude for work abilities of PWE, problems at school, fulfilment of life goals as a reason for “end of seizures”, established inability to perform a profession, possibility of retraining, termination of employment due to illness, retirement-lifestyle change, disclosure of illness to employees, severe prejudice of potential employer, prejudice of medical staff, the birth of a child – reason for job loss) - RELATIONSHIPS (separation from partner, partner's desire for a child, singleness, husband's support, change in relationships due to illness, mother's influence on life with disease, awareness of involvement in family and work, honesty towards potential partner) - PWE LIVING ENVIRONMENT (poorer quality of life, understanding of other PWE regarding hiding the disease, feeling different due to illness, care from relatives, family support, limitations in daily tasks, safety net, financial compensation, seizure while driving, accepting the disease, giving up cycling, less work on a computer, introducing new habits in life, carefree – changing perceptions of things, making sense of a changed future, finding reasons for the disease, giving up alcohol – impact on social life, revoked driver's license, adapting to the disease improves quality of life, the need for a stress-free life, spontaneous acceptance of the future) |
| **Cause of stem narrative** | - EMPLOYMENT (financial hardship reason for not continuing education, employment – reason for hiding the disease, refusal to employ PWE, restriction of work ability, disagreement with legal restrictions for PWE, need to differentiate PWE according to the form of epileptic seizure, honesty towards potential employer, requirement to disclose medical condition prior to taking up duty |
| **Stem narrative’s connection to relationships** | - ROMANTIC RELATIONSHIPS (impact of epilepsy on relationships, illness as a reason for divorce, seeking contacts for relationships, planning a future together, fear of having a relationship with PWE, development and personal growth in the relationship, the burden of illness for the wife, the woman's involvement in lifestyle changes, awareness of acceptance of the disease by the husband) - FAMILY (knowledge of the impact of the disease on children, good relationship with the environment, parents 'responsibility in raising children, sister's care for the brother-in-law, dependence on relatives, awareness of relatives' burden, safety net, encouragement and support from husband, parents’ care, parents’ disappointment because daughter with epilepsy is not schooled, comparison to siblings, fear of wife and child of driving with PWE, mother's influence, facing relatives with the consequences of illness, help of family members, neglecting relationship with wife and children, benefits of loneliness, the importance of family relationships) - RELATIONSHIP WITH EMPLOYER AND CO-WORKERS (ridicule from employees during the seizure, sympathy from (only) two employees, non-understanding and harassment of older co-workers, desire for a better working environment, experiencing unequal treatment when looking for a job, absence from work due to the consequences of a seizure, proposal to raise awareness of public service employees, hiding illness from co-workers, ignorance of illness limitations, stigma among co-workers) - RELATIONSHIP WITH CLASSMATES, TEACHERS AND FRIENDS (acceptance from friends, encouragement from classmates, own experience of illness as encouragement for other patients, prejudice against healthy people, ridicule from teachers and classmates, distrust of others about PWE’s abilities, lack of interest in others, concealing the disease from friends) |
