# Supplementary material for: Psychosocial Factors in the Experience of Epilepsy: A Qualitative Analysis of Narratives
Source: Behav Neurol. 2021 Jul 26;2021:9976110. doi: 10.1155/2021/9976110 (PMC8331301; doi:10.1155/2021/9976110)
Supplement: Supplementary Materials — Appendix 1: sociodemographic data of PWE included in the study. The table shows the PWE data included in the survey. We collected data on the age of PWE, marital status, environment in which PWE lives, education, employment, age at the first epileptic seizure, time elapsed since the last epileptic seizure, and whether PWE is included in the League Against Epilepsy of Slovenia. Appendix 2: subcategories and their associated codes by criteria. The table shows the individual criteria and their subcategories. We included in the subcategories the codes that we defined during coding. Subcategories with codes show the content of each criterion. Appendix 3: number of codes by stem narrative for each criterion. The table shows the number of codes in each criterion according to each stem narrative. The number of codes indicates the frequency of their occurrence in a particular criterion. [file 9976110.f1.zip › Appendix 3_Number of codes by stem narrative for each criterion.docx]

Appendix 3: Number of codes by stem narrative for each criterion

|  | **Stem narrative** | **Emotional aspect of stem narrative** | **Personal engagement** | **Consequences of stem narrative** | **Cause of stem narrative** | **Stem narrative's connection to relationships** | **Repetitions** |
| --- | --- | --- | --- | --- | --- | --- | --- |
| 1. | Disease changes the way you see yourself and others | 4 | 10 | 9 | 0 | 8 | 5x |
| 2. | Burden of disease in a relationship | 5 | 5 | 5 | 0 | 7 | 3x |
| 3. | Unemployment due to disease limitations | 5 | 4 | 4 | 1 | 5 | 4x |
| 4. | Loss of independence and burden of relative’s care | 9 | 2 | 11 | 0 | 6 | 6x |
| 5. | Non-understanding of the burden of disease at school and work | 7 | 5 | 8 | 1 | 9 | 9x |
| 6. | Husband’s relationship and role | 7 | 1 | 6 | 0 | 5 | 5x |
| 7. | Giving up sports and separation from partner | 8 | 4 | 4 | 0 | 2 | 4x |
| 8. | Alcohol addiction | 1 | 2 | 7 | 0 | 2 | 4x |
| 9. | Limited work ability and effort in performing work | 4 | 6 | 5 | 0 | 8 | 9x |
| 10. | Limited work ability and retirement problems | 4 | 4 | 5 | 6 | 2 | 4x |
| 11. | Giving up further education | 6 | 6 | 7 | 0 | 4 | 3x |
| 12. | Loneliness | 11 | 5 | 3 | 0 | 6 | 4x |
| 13. | Hiding the disease | 1 | 1 | 1 | 1 | 2 | 1x |
| 14. | Disease lifestyle adjustment | 7 | 7 | 7 | 0 | 5 | 5x |
| 15. | Accepting the disease and lifestyle changes | 12 | 15 | 9 | 0 | 8 | 6x |
| 16. | Lifestyle changes due to the disease | 6 | 7 | 7 | 0 | 6 | 3x |
| 17. | Career change | 9 | 6 | 6 | 0 | 2 | 5x |
| 18. | Physical hazard during an epileptic seizure | 9 | 1 | 6 | 0 | 4 | 4x |
| 19. | Difficult family situation | 3 | 0 | 1 | 0 | 1 | 2x |
| 20. | Driving license | 8 | 4 | 7 | 0 | 2 | 4x |
| 21. | Initial concealment and subsequent disclosure of the disease | 3 | 3 | 4 | 0 | 5 | 3x |
